# Effects of Chip Type on the Properties of Chip–Sawdust Boards Glued with Polymeric Diphenyl Methane Diisocyanate

**DOI:** 10.3390/ma13061329

**Published:** 2020-03-14

**Authors:** Radosław Mirski, Adam Derkowski, Dorota Dziurka, Marek Wieruszewski, Dorota Dukarska

**Affiliations:** Department of Wood Based Materials, Faculty of Wood Technology, Poznań University of Life Sciences, Wojska Polskiego 38/42, 60-627 Poznań, Poland; rmirski@up.poznan.pl (R.M.); marek.wieruszewski@up.poznan.pl (M.W.); dorota.dukarska@up.poznan.pl (D.D.)

**Keywords:** mechanical properties, chip geometry, chips, sawdust, chip–sawdust board

## Abstract

This study investigates the effects of chip type and sawdust percentage on physical and mechanical properties of chip–sawdust boards. The used wood chips varied in linear dimensions and original source. The origin determined the wood quality, which translated into the chips’ linear dimensions. The used materials were chips from sawmill waste processing, aggregate processing of sawmill wood, and chips intended for medium-density boards. The experiment demonstrated that the best boards in terms of mechanical properties were obtained from 4-mm-thick chips with 30% sawdust content and a density of 850 kg/m^3^. These boards meet the requirements of the EN 312 (2010) standard for P5 boards.

## 1. Introduction

Wood-based materials are mostly made from wood-derived products, such as particles or strands joined with glue. Their properties depend on the quality of the used particles or strands and, more specifically, on their linear dimensions. The particles’ geometry determines the mechanical properties of wood-based materials, while the adhesive determines their resistance or durability under specific process conditions. Wood-based materials are also classified depending on the particle size and orientation. The main goal of machining is obtaining appropriate material for further production, i.e., retaining as many wood characteristics as possible, which is usually related to particle geometry [1,2,3]. The linear dimensions of wood particles considerably affect the mechanical and physical properties of the boards made from them [4,5,6].

The desired dimensions of the particles are obtained via careful machining, which generates chips of specific linear dimensions, primarily thickness. The other linear dimensions are less controllable. While controlling length may be, to a certain degree, regulated by the length of the raw materials or special cutting knives, chip width is quite often the result of uncontrolled breaking. With lower requirements for the final product, a lower-quality (shape, linear dimensions) material can be used to produce chips intended for manufacturing wood products. Currently, the minimum requirements for particleboards containing fine chips in this respect are set in Europe by the EN 312 standard [7]. Boards of this type can be successfully produced from various types of biomass, e.g., fragmented primary (forest) wood [8,9], secondary wood [10], or crop straw [11,12,13]; all types of resins, pure, modified, or hybrids, may be used to bond them [14,15,16,17,18,19]. Irrespective of the wood’s origin, it is first fragmented into larger pieces, 30 to 60 mm in size and 3 to 9 mm in thickness (chips), and then into proper strands, 10 to 20 mm long, 3 to 10 mm wide, and 0.4 to 0.7 mm thick.

A considerable portion of the chips used by particleboard facilities is generated by sawmills. Chips result from the fragmentation of wood pieces formed during the production of lumber with set dimensions, as well as the fragmentation of low-quality lumbers or wings. Processing round-wood in cants with the use of chipping canters is also becoming popular. The different methods of chip production and the types of fragmented wood are the main reason for the various linear dimensions of wood chips. Therefore, regardless of the origin of the chips and their production method, they need to be further fragmented into strands to unify their geometry to meet technological requirements. Sawdust, produced by other facilities than sawmills alone, is sorted and shredded, if necessary, and then combined with chips to produce standard particleboards. As some of the produced boards are relatively thick, i.e., over 22 mm, they may be made from coarser materials than those commonly used. Using coarser materials also allows for saving energy spent on obtaining the final material used for mat formation.

This study investigated the option of using wood materials generated in various sawmill facilities without further processing for chip–sawdust board production. As the moisture content of wood chips and sawdust stored in the open air can vary by up to about 70% over the course of a year, the drying process was limited by using an adhesive that allows for gluing damp wood.

## 2. Materials and Methods

This study involved three types of wood chips and pine sawdust (*Pinus Sylvestris* L.). The first type of chips was obtained via fragmentation of side surfaces of timber wood with a chipping head; the second type was obtained via fragmentation of sawmill waste in a chopper for further processing in papermaking or the particleboard industry; and the third type was obtained via fragmentation of wood in a chopper (Research & Development Centre for Wood-Based Panels, Czarna Woda, Poland) for the production of dry fiberboards. The fraction remaining after elimination of particles that were too large (on a 50 mm × 50 mm flat screen) underwent a dimensional analysis. Sawmill chips (type A) were 36.3 mm long, 12.7 mm wide, and 4 mm thick. Head chips (type B) were 30.3 mm long, 11.8 mm wide, and 5.0 mm thick, while defibered chips (type C) were 33.3 mm long, 21.2 mm wide, and 7 mm thick. These specified linear dimensions represent about 100 chips collected randomly from a prepared mass of chip samples. These samples were not identified based on the chips’ origin, but based on the type of chopper and setting of knives.

**Figure 1:**
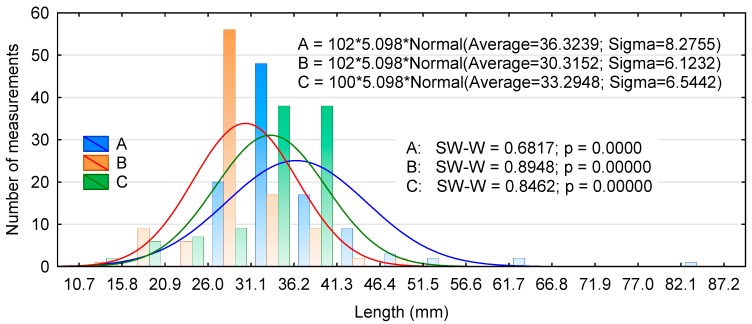
Histogram of the chip length distribution for two production batches.

**Figure 2:**
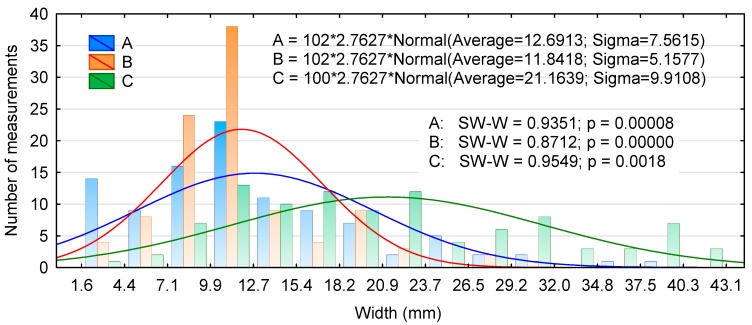
Histogram of the chip width distribution for two production batches.

**Figure 3:**
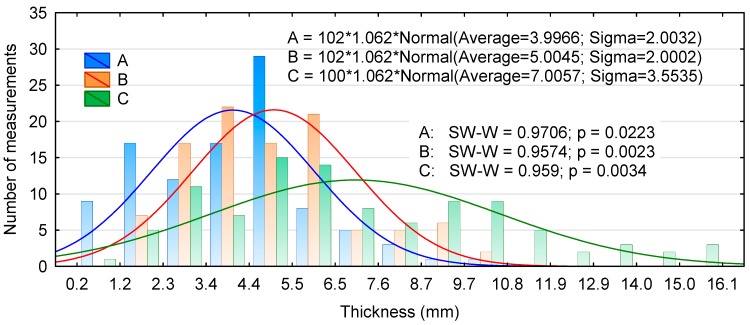
Histogram of the chip thickness distribution for two production batches.

The distribution of linear dimensions of the experimental chips is presented in Figure 1, Figure 2 and Figure 3. Figure 1 shows that the lengths of types A and B chips are close to the average, while as many as 75% of type C chips have lengths of 31.1 to 36.2 mm and 36.2 to 41.3 mm. Moreover, none of the distributions were normal. Similarly, no normal distribution was achieved for the width of the chips, irrespective of the production method (Figure 2). In addition, chips generated with the chipping head were predominantly between 8.9 and 11.3 mm in width; this accounted for nearly 40% of the total chip mass. The lower average thickness of type A chips was due to the presence of a larger number of thinner chips. Many of the type A and B chips fell into the middle of the thickness distribution interval, while type C chips showed a large variability over the thickness range (Figure 3). The Kruskal–Wallis test (marked as SW-W) revealed that the chips only differed significantly in their thickness (Table 1). Type A and C chips had statistically similar lengths and type A and B chips had similar widths.

Before gluing, the wood chips with a moisture content of 21% were mixed with pine sawdust with a moisture content of 18%. The sawdust content was 30% or 50% of the chips’ dry weight. The mixture was glued with polymeric diphenyl methane diisocyanate (pMDI, ONGRONAT® 2100, BorsodChem Group, Kazincbarcika, Hungary); 4% pMDI was used per dry weight of total mass. One-ply boards were pressed to reach an assumed thickness of 26 mm and a density of 650 kg/m^3^, 750 kg/m^3^, and 850 kg/m^3^. The temperature of the heating plates was 200 °C. The maximum pressure reached 2.5 MPa for the boards with a density of 650 kg/m^3^, 2.8 MPa for the boards with a density of 750 kg/m^3^, and 3.5 MPa for the boards with a density of 850 kg/m^3^. The mat was pressed for 25 s/mm of the board thickness; the total time of press closing and opening was 30 s.

**Figure 4:**
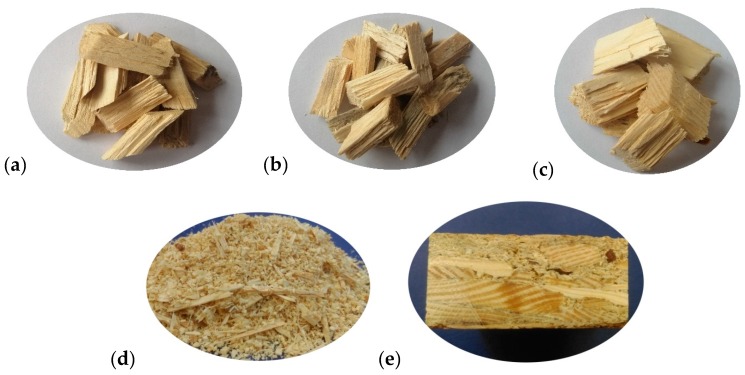
Appearance of the raw wood materials used to make the boards and the final board: (**a**) Type A chips; (**b**) Type B chips; (**c**) Type C chips; (**d**) Sawdust; (**e**) Chip–sawdust board.

Variants of the boards are presented in Table 2, and the appearance of the raw materials and final board is shown in Figure 4.

After an acclimation (seven days, 55 ± 5% RH, 21 ± 1 °C), the boards were tested as per the relevant standards and the following parameters were assessed:

- modulus of rupture (MOR) and modulus of elasticity (MOE) according to EN 310 [20];
- internal bond (IB) according to EN 319 [21]; and
- thickness swelling (TS) after 24 h according to EN 317 [22] and water absorption (WA).

The assessments of mechanical properties and water resistance involved 6 to 12 samples of each variant. The remaining analyses were made in three to five replications. The results were analyzed using the STATISTICA 13.0 package (Version 13.0, StatSoft Inc., Tulsa, OK, USA).

## 3. Results and Discussion

Table 3 presents the properties of the experimental chip samples and chip–sawdust boards evaluated using a three-point bending test. They had a relatively high modulus of elasticity of over 2300 N/mm^2^ at a coefficient of variation below 8.5%. The modulus of rupture (MOR) showed a considerably greater variability, which was probably due to the uneven distribution of sawdust among the chips. Considering the adhesive, the boards should meet the requirements of the EN 312 (2010) standard for P5 and P3 boards. The statistical analysis demonstrates that the boards meet the requirements for the MOE irrespective of the chip quality, sawdust percentage, or density. In terms of bending strength, only the boards of higher density made from type A chips were acceptable. The three-factor ANOVA for the main factors showed that the boards differ significantly in their bending strengths and moduli of elasticity depending on the type of chips, density, and sawdust percentage (small letters in Table 3 of the first three columns).

The assumed density intervals were 100 kg/m^3^, which ensured considerable differences in the boards’ properties. The increasing sawdust content negatively correlated with the bending strength and MOE. Moreover, as the chips only differed in thickness (Table 1), the changes strongly depended on thickness, rather than other linear parameters. The findings corroborated a well-known observation of reduced bending strength above a certain chip thickness. The individual board variants belong to numerous homogeneous groups identified in Tukey’s test. This classification is likely due to various effects of the chips’ type and the percentage of sawdust on the MOR and MOE values. Tukey’s test was performed for one-factor analysis. It demonstrated no clear effects of the content of sawdust when in the 30% to 50% range. Statistically, better properties were evaluated in the bend test for boards made from type A chips and a 30% content of sawdust than for boards made of type C chips and the same sawdust percentage. Although the performed tests did not allow for a determination of relations for the three variables, the discussed properties showed a clear linear dependence on the chips’ thickness, which confirmed the much stronger effect of the chip type than of the sawdust percentage (Table 4).

The internal bond for the 26-mm-thick P3 and P5 boards was 0.35 N/mm^2^. This condition was met by chip boards with 30% sawdust content made from type A chips with densities of 750 kg/m^3^ and 850 kg/m^3^ and from type B chips with a density of 850 kg/m^3^ (Table 5).

The condition was almost met by the boards with an even percentage of chips and sawdust, but the value of the fifth percentile, at only 0.32 N/mm^2^, was too low. However, the three-factor analysis revealed no differences in IB caused by different percentages of chips and sawdust. Similarly, the result of the post hoc Tukey’s test was 0.7997, and the result of the two-factor analysis without chip-only boards was F (1, 80) = 0.08235, *p* = 0.77488. Therefore, while the effects of density are obvious at this sawdust percentage, the chip type is also important. Moreover, this study confirmed that the mechanical properties of chip–sawdust boards and chip boards depend primarily on the chip quality.

The thickness swelling of the chip–sawdust boards was relatively high (Table 5). This was likely due to not having used additional agents for improving hydrophobicity and utilizing only highly coarse material, i.e., large pieces of wood. Swelling was most intense for the boards made only from chips, and more so for type B and type C chips. The three-factor analysis that did not take chip-only boards into account showed no significant differences in the swelling of the boards made from type B and type C chips; the latter had swelling that was about 50% greater than boards made from type A chips. Moreover, the higher the sawdust content, the lower the board swelling. This is likely due to the lower swelling of the finer fractions that fill in spaces between larger pieces of wood.

**Figure 5:**
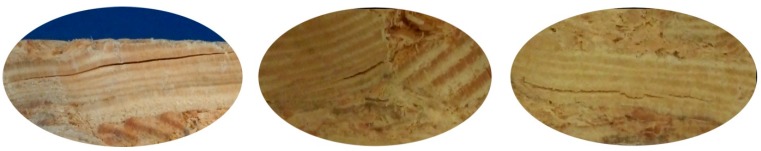
Cracked chips in the board structure.

Tests indicated that the mechanical properties of the chip boards strongly depend on chip quality, and more specifically on chip thickness. The chip and strand thickness restrict the number of board layers. As the board components become larger, fewer layers can be formed, which results in lower mechanical properties. A beneficial effect of increasing board thickness with a constant gluing degree, as described in this study, is the decrease in the specific area of the glued material. This results in an improved loading coefficient, defined as the ratio of the adhesive’s dry weight to the specific surface of the glued material. This effect was noted in the study. However, large chips may crack during water-soaking and when subjected to the tensile test (Figure 5). Under these conditions, the chips split and the resulting values are considerably lower than the average value. In the water-soaking case, this effect is likely caused by desorption changes or stress. In the IB case, chip cracking was only sporadic and occurred mostly in low-density boards. The tensile bond of pine wood ranges between 2.9 N/mm^2^ and 4.2 N/mm^2^, depending on the quality of the wood used for chip preparation and its anatomical arrangement. This value is about 10 times greater than the board IB, which means that there must be other reasons for chip cracking. In this case, it was probably due to the low quality of bonding associated with insufficient pressing and insufficient contact between the board’s components. Furthermore, chip cracking occurred in boards not subjected to water-soaking or the tensile test. This may have been be caused by desorptive stress, as the boards were made from material with a relatively high moisture content, and by stress generated during the mat pressing.

## 4. Conclusions

Unprocessed sawmill by-products in the form of chips and sawdust may serve as materials of full value for the production of wood-based boards with favorable physical and mechanical properties. Such boards have a limited minimum thickness, starting from approximately a dozen millimeters, a higher density of about 50 to 100 kg/m^3^, and allow for the use of fragmented wood material generated in sawmill facilities.

Boards with the best properties contained chips of lower thickness and a percentage of sawdust of up to 50%. Although the study did not demonstrate it clearly, it seems that a combination of chips and sawdust positively affects the board quality by making its structure more homogeneous.

The chips and sawdust should be thoroughly mixed to limit the presence of free spaces in the board structure, which lowers its strength. The mechanical properties are also decreased by individual chip cracking; the latter’s cause is not entirely clear.

The best boards in terms of mechanical properties were obtained from type A chips with a 30% content of sawdust and a density of 850 kg/m^3^.

## Figures and Tables

**Table 1:** The Kruskal–Wallis Test and ANOVA of Kruskal–Wallis Ranks.

Parameter	Average Rank A	Average Rank B	Average Rank C	H	p	A/B	A/C	B/C
Length (mm)	183.3529	102.6078	171.9200	50.3089	0.0000	6.5600	0.9242	5.6032
Width (mm)	129.9118	118.8608	209.7500	64.0052	0.0000	0.8896	6.4541	7.3394
Thickness (mm)	113.2206	149.2647	195.8650	44.8455	0.0000	0.0102	0.0000	0.0005

**Table 2:** Variants of Experimental Chip Boards.

Chip Type	A			B		C
Sawdust percentage (%)	0	30	50	30	50	30
Density (kg/m^3^)	-	650	-	650	-	-
	750	750	750	750	750	750
	-	850	-	850	-	-

**Table 3:** Particleboard Properties Yielded by the Three-Point Bending Test.

Chip Type	Sawdust Percentage (%)	Board Density (kg/m^3^)	MOR ***		MOE ****	
			x (N/mm^2^)	CV ** (%)	x (N/mm^2^)	CV ** (%)
A ^c^*	0 ^c^	750 ^b^*	18.0 ^e^*	6.6	3570 ^d^*	2.9
A ^c^	30 ^b^	650 ^a^*	11.4 ^a,b^	2.0	2640 ^a,b^	1.6
A ^c^	30 ^b^	750 ^b^	14.1 ^b,c,d^	7.9	3250 ^c,d^	4.3
A ^c^	30 ^b^	850 ^c^	17.1 ^d,e^	7.6	4190 ^e^	1.6
A ^c^	50 ^a^	750 ^b^	13.4 ^a,b,c^	7.8	3390 ^d^	4.2
B ^b^	30 ^b^	650 ^a^	10.7 ^a,b^	17.5	2430 ^a^	8.4
B ^b^	30 ^b^	750 ^b^	12.8 ^a,b,c^	15.9	3000 ^b,c^	8.0
B ^b^	30 ^b^	850 ^c^	15.5 ^c,d,e^	4.1	3620 ^d^	3.7
B ^b^	50 ^a^	750 ^b^	11.4 ^a,b^	4.5	2750 ^a,b^	2.6
C ^a^	30 ^b^	750 ^b^	10.1 ^a^	12.2	2380 ^a^	4.4

^a,b,c,d,e^* Letters denote homogeneous groups and consecutive letters denote increasing values. ** Coefficient of variation; *** Modulus of rupture; **** Modulus of elasticity.

**Table 4:** The Percentage Effect of Factors on Static Bending Strength and Modulus of Elasticity (MOE).

Factor	Bending Strength		MOE	
	Sum of Squares	Percentage Effect of the Factor	Sum of Squares	Percentage Effect of the Factor
Chip type	29.405 (29.405) ^1^	10.3 (14.4)	2,635,961	22.8
Board density	112.101 (112.101)	39.2 (55.0)	7,576,108	65.5
Sawdust percentage (%)	78.360 (4.569)	27.4 (2.2)	285,600	2.5
Error	60.070 (57.732)	23.1 (28.4)	1,071,187	9.2
Total	285.936 (203.807)	100.0	11,568,856	100

^1^ Values in parentheses result from an analysis that does not account for chip-only boards.

**Table 5:** Internal Bond (IB) and Thickness Swelling (TS).

Chip Type **	Sawdust Percentage ** (%)	Board Density ** (kg/m^3^)	IB		TS	
			x (N/mm^2^)	CV *** (%)	x (N/mm^2^)	CV (%)
A ^c^*	0 ^b^*	750 ^b^	0.50 ^f^*	13.6	41.9 ^g^*	9.4
A ^c^	30 ^a^*	650 ^a^	0.30 ^a,b,c^	13.0	21.5 ^b^	4.8
A ^c^	30 ^a^	750 ^b^	0.41 ^d^* ^,e^*	11.2	17.1 ^a^	7.6
A ^c^	30 ^a^	850 ^c^	0.52 ^f^	13.9	26.5 ^c^	16.9
A ^c^	50 ^a^	750 ^b^	0.36 ^b,c,d^	6.55	19.4 ^a,b^	8.5
B ^b^	30 ^a^	650 ^a^	0.27 ^a^	10.2	27.6 ^c,d^	12.6
B ^b^	30 ^a^	750 ^b^	0.33 ^a,b,c^	4.46	34.7 ^e,f^	2.7
B ^b^	30 ^a^	850 ^c^	0.48 ^e,f^	8.24	36.8 ^f^	3.4
B ^b^	50 ^a^	750 ^b^	0.37 ^c,d^	7.71	31.1 ^d,e^	2.8
C ^a^	30 ^a^	750 ^b^	0.29 ^a,b^	17.4	32.6 ^e^	6.6

^a,b,c,d,e,f,g^* Letters denote homogeneous groups and consecutive letters denote increasing values. ** Values from a three-factor analysis in Tukey’s honest significant difference (HSD) test for IB. *** Coefficient of variation.
